# Effect of Different Cooking Methods on Nutrients, Antioxidant Activities and Flavors of Three Varieties of *Lentinus edodes*

**DOI:** 10.3390/foods11172713

**Published:** 2022-09-05

**Authors:** Xiaoli Zhou, Qinglin Guan, Yanli Wang, Dong Lin, Bin Du

**Affiliations:** School of Food and Pharmaceutical Engineering, Guiyang University, Guiyang 550005, China

**Keywords:** cooking method, flavor, nutrients, antioxidant activity, volatile, *Lentinus edodes*

## Abstract

This work evaluated the effect of different cooking methods (boiling, steaming, microwaving, frying and pressure cooking) on the nutrients, antioxidant activities, volatile and nonvolatile taste-active components of three varieties of *Lentinus edodes* (808, 0912 and LM) from Guizhou Province. The results showed that LM had the most polysaccharides, 0912 had the most minerals, but LM, 808 and 0912 had low amounts of polyphenols, dietary fiber and proteins, respectively. The dietary fiber and protein were decreased by 4.1~38.7% and 4.1~44.0% during cooking, while microwaving improved the nutritional value of the *Lentinus edodes* by increasing the polysaccharide (88~103 mg/g to 93~105 mg/g) and polyphenol content (6.4~8.1 mg/g to 7.5~11.2 mg/g), thereby strengthening the antioxidant activity. The nucleotides were all destroyed after cooking, especially frying or boiling. The glutamate content was the highest in LM and 808, and the methionine content appeared to be the highest in 0912. Pressure cooking and frying increased the proportions of sweet and umami amino acids and decreased the proportion of bitter amino acids, creating more aroma-active compounds. In summary, microwaving increased the content of bioactive compounds and antioxidant activities, and it preserved nonvolatile taste-active components, while pressure cooking and frying were the best methods for increasing the flavor compounds.

## 1. Introduction

The edible mushroom is a traditional vegetable in China and part of human diets around the world. It has a unique and subtle flavor, high levels of proteins and dietary fibers, low levels of fats, and functional properties [1]. Recent reports indicate that edible mushroom extracts exhibit promising therapeutic and health-promoting benefits, particularly in relation to diseases associated with inflammation [2], anticancer activities, cholesterol-lowering effects and antioxidant, anti-atherosclerotic, antihypertensive and anti-aging properties [3,4,5]. 

China is rich in mushrooms. China’s edible mushroom output ranks first in the world. Among the mushrooms, *Lentinus edodes* (shiitake) is a kind of edible fungus widely distributed, mainly from Zhejiang Province, Guizhou Province and Fujian Province. China was the first country to grow *Lentinus edodes* (LE), with a history of more than 800 years. LE contains all the essential amino acids, polysaccharides and polypeptides, and it is a rich source of minerals such as potassium, zinc and iron [6,7]. It used as a “functional food” for the treatment of tumors, heart diseases, high blood pressure, obesity, diabetes, liver ailments, exhaustion and weakness [8,9,10]. Generally, LE is consumed as dried mushrooms, canned, in soup, as seasoning, as pickles and in other products.

Cooking can improve food’s taste and flavor and promote its digestion and absorption by the human body [11]. Some of the existing nutrient content may be decreased or enhanced, and new nutrient and anti-nutrient compounds are also formed during cooking. Thermal treatments have been known to affect the nutritional value, chemical composition and texture of food. The effects of cooking on different species of mushrooms and other foods have been studied; for example, Sun et al. [12] studied the effects of domestic cooking methods on the nutrient, phytochemical and flavor contents of mushroom soup. Tan et al. [13] reported that the enhancement or reduction of the polyphenols and antioxidant activities of oyster mushroom soup depended on the cooking method and mushroom variety. Chang et al. [14] studied the effects of domestic cooking methods on the total phenolic contents, antioxidant activities and sensory characteristics of *Hericium erinaceus*. Chiocchetti et al. [11] reported the effect of cooking on the contents of toxic trace elements in dried mushrooms. These results showed that each method had a specific impact on the properties of the food. The phytochemical contents, microorganisms and anti-nutritional factors varied according to the mushroom species, nutritional composition and physical or chemical reactions.

At present, the research on mushrooms mainly focuses on extracts and functions [15,16,17]. Nevertheless, flavor is an important indicator for mushroom products. The characteristic flavor substances of mushrooms can be classified into volatile (smell) and nonvolatile components (taste). The particular tastes of some mushroom species are primarily attributed to several water-soluble compounds, including soluble sugars, free amino acids and 5′-nucleotides [15,16,17]. Many volatile flavor compounds can increase the attractiveness of food by stimulating consumers’ sense of smell. With the development of science and technology, from the initial sensory analysis to further qualitative and quantitative analyses of flavor components, mushrooms’ flavors have come to be more deeply studied. However, studies on the flavors of mushrooms after cooking are limited compared to those on the flavors of broth made from animal materials, such as Chinese Piao chicken meat [18], chicken breast [19] and squid mantle muscle [20]. Moreover, there has been no extensive investigation into the effect of different cooking methods on different LEs’ nutrient and flavor characteristics, which will further influence the product. In particular, there is a need for systematic studies on LEs’ physicochemical properties and flavor-related compounds (nonvolatile and volatile components). There is still a lack of conclusive and comprehensive reports on LE from Guizhou Province, China, perhaps because of geographic and climatic limitations. Therefore, the objective of this study was to evaluate the effects of different cooking methods (microwaving, boiling, steaming, pressure cooking and frying) on the nutrients, antioxidant activities and flavors of three varieties of LE from Guizhou Province, to provide a scientific and in-depth reference for the further processing of different kinds of Chinese LE.

## 2. Materials and Methods

### 2.1. Lentinus edode Samples and Cooking Methods

Fresh fruiting bodies of three kinds of *Lentinula edodes*—0912, 808 and LM—were harvested from cultivation rooms in Guizhou Province, China. The fresh mushrooms’ fruiting was cleaned of soil and substrates. The samples were cut to be 1 × 1 × 0.5 cm thick, and were randomly divided into six groups. One of them remained raw, and the rest were cooked using one of five methods (boiling, microwaving, frying, steaming or pressure cooking).

The cooking conditions were performed according to the method by Sun et al. [12], Lee et al. [21] and Irene et al. [22] with minor adjustments; the experiment was carried out in triple.

1. Boiling (BO): LE was boiled in 1:10 boiling water for 10 min using an induction cooker (Joyoung Ltd., Jinan, China).
2. Frying (FR): LE was fried in a pan with rapeseed oil (160 °C) for 2 min using an induction cooker (Joyoung Ltd., Jinan, China).
3. Microwaving (MI): LE was placed in a dish and cooked in a microwave oven (Midea Ltd., Guangdong, China) at 800 W for 3 min.
4. Steaming (ST): LE was steamed in 1:10 boiling water for 10 min.
5. Cooking at high pressure (HP): LE was treated in an autoclave (Boxun Co., Ltd., Shanghai, China) at 110 °C for 15 min.

After cooking, all the samples were placed on filter paper to drain the excess water or oil. The raw and processed LE was freeze-dried (Songyuan Huaxing, Beijing, China), powdered and homogenized for subsequent experiments.

### 2.2. Nutrient Components

The chemical composition of the LE, including the moisture, ash, polysaccharide, dietary fiber and protein, was determined in triplicate according to Association of Official Analytical Chemists methods (1995) [23]. The dietary fiber was determined by the enzyme gravimetric method. The protein content was determined using the Kjeldahl method with a conversion factor of 6.25. The moisture content was determined by heating the fresh sample at 105 °C overnight until it reached a constant weight. The ash content was determined by weighing the residue obtained after incineration at 550 °C in order for it to reach a constant weight. The polysaccharide content was determined using the phenol–sulfuric acid method [24]; a curve was prepared using a standard solution of glucose (Y = 1.0594x + 0.0603, R^2^ = 0.9992). All the other chemicals and solvents were analytical grade and obtained from Sinopharm Chemical Reagent Co., Ltd. (Shanghai, China). 

*Total phenolic content analysis.* Freeze-dried mushroom powder (1.0 g) was triple-extracted with 30 mL of 60% (*v/v*) ethanol in an ultrasound bath (100 W) for 30 min. After centrifugation at 6000 rpm for 15 min, the supernatants were combined and adjusted to 100 mL for the measurement of the total phenolic content (TPC), which was determined by the Folin–Ciocalteu method [21,25]. Briefly, 0.5 mL of mushroom extract was added to a 25 mL colorimetric cylinder containing 10 mL of water and 0.5 mL of Folin–Ciocalteu reagent, and then mixed well. After 5 min, 5 mL of 5% Na_2_CO_3_ solution was added and mixed in with a vortex shaker, using distilled water to adjust the total volume to 25 mL. After 60 min, the absorbance at 750 nm was measured in a UV-2550 spectrophotometer (Shimadzu Co., Kyoto, Japan) using distilled water as a blank. A calibration curve was prepared using a standard solution of gallic acid (Y = 0.6749x + 0.0711, R^2^ = 0.9994).

### 2.3. Antioxidant Assays

*Preparation of ethanol extracts.* Ethanol extracts were prepared as described by Nie et al. [9] with minor modifications. First, 1 g of the sample was placed in a tube and 15 mL of 60% ethanol was added. The tube was thoroughly shaken ultrasonically at room temperature for 30 min and centrifuged at 6000 rpm for 15 min at 4 °C, and the supernatant was recovered. We repeated the extraction three times. The extracts were then combined and the supernatant was diluted to 50 mL.

*DPPH-scavenging activity.* Radical-scavenging activity was estimated following the procedure reported by Liu et al. [1] and Anuduang et al. [25]. Briefly, 2 mL of the sample was mixed with 2 mL of 0.2 mM DPPH ethanol solution. After incubation for 30 min, the absorbance was measured at 517 nm using the same plate reader.

*Reducing power.* The reducing power of the ethanol extracts was determined using the method presented by Nie et al. [9]. About 2.5 mL of 1% freshly prepared potassium ferricyanide solution was mixed with 1 mL of the sample and 2.5 mL of 50 mM phosphate buffer solution (pH 6.6). The mixtures were incubated at 50 °C for 20 min. At the end of the incubation, 2.5 mL of 10% trichloroacetic acid was quickly added to the mixtures, followed by centrifugation at 3000 r/min for 10 min at 4 °C. Finally, 2.5 mL of supernatant was mixed with 2.5 mL of distilled water and 0.5 mL of 0.1% ferric chloride; the absorbance was measured at 700 nm in the same plate reader.

### 2.4. Flavoring Substances

#### 2.4.1. Nonvolatile Compound Analysis

**Figure 1:**
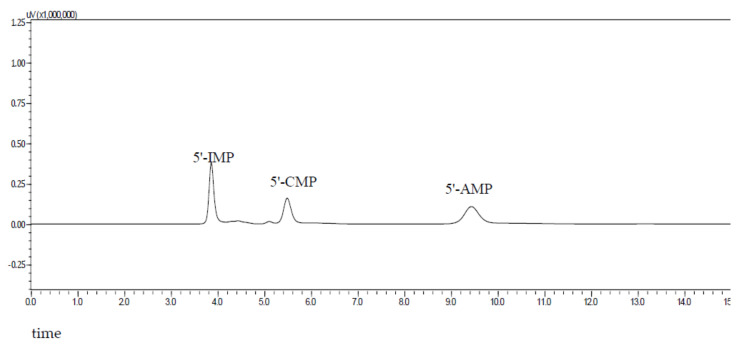
HPLC chromatogram of three nucleotide standards.

*Nucleotide assay.* Nucleotides were extracted as described by Sun et al. [12] with minor modifications. Freeze-dried sample powder (1.0 g) was extracted using 5 mL of distilled water and boiled for 5 min. Then, it was cooled to 4 °C, centrifuged at 8000× *g* for 15 min and then filtered using a 0.22 μm water-system filter membrane for HPLC analysis (Agilent 1100, New York, NY, USA). The nucleotides were analyzed using a WondaSil C18 column (4.6 mm × 250 mm, 5 μm). The mobile phase was a 0.01 M KH_2_PO_4_ buffer solution (A) and methanol (B), their ratio was 8:2, and the flow rate was 1 mL/min. The column temperature was 25 °C, and equal-gradient elution was performed. The chromatograms of the three nucleotide standards are shown in Figure 1. Each nucleotide was identified using the standard compound and quantified.

*Free amino acid analysis.* Amino acid composition was analyzed according to the reports of Sun et al. [12] and Liu et al. [6]. First, 1.0 g of freeze-dried sample powder was diluted with 25 mL of trichloroacetic acid (5%, *v*/*v*) and incubated for 20 min. This was then filtered with double-layered filter paper and centrifuged at 10,000 rpms for 30 min. This sample, as described above, was filtered through a 0.22 μm water-system filter membrane (Shanghai Xingya Purification Material Co., Shanghai, China). The concentrations of free amino acids were determined by an automatic amino acid analyzer (Agilent 1100 Series, Palo Alto, CA, USA) in the School of Food Science and Technology, Jiangnan University. Amino acids were precolumn derivatized with o-phthalaldehyde and 9-fluorenylmethyl chloroformate. Mobile phase A contained 0.8% sodium acetate, 0.5% tetrahydrofuran and 0.0225% triethylamine (pH 7.2); mobile phase B was prepared as follows: 400 mL 2% sodium acetate–acetic acid solution (pH 7.2) were mixed with 800 mL acetonitrile and 800 mL methanol. The gradient elution was as follows: the initial 92% of A was gradually decreased to 40% within 27.5 min, and then further decreased to 0 within 4 min. After holding for 2.5 min, mobile phase A was returned to 92% again within 1.5 min. The peak identification and quantification from the instrument software adhered to FAA standards (Sigma Chemical Co. St. Louis, MO, USA). 

#### 2.4.2. Volatile Compound Analysis

The volatile compounds in LE were determined according to a previous study with minor modifications [26]. The volatiles were extracted by headspace solid-phase microextraction (HS-SPME), placing 2.0 g of the sample in a 20 mL headspace glass sampling vial. An SPME fiber (DVB/CAR/PDMS) (Supelco, Bellefonte, PA, USA) was used to extract the volatiles at 60 °C for 30 min. Afterward, an HS-SPME fiber was inserted into the injection port of the gas chromatography–mass spectrometry (GC/MS) system for thermal desorption.

The GC/MS was performed using a Trace GC and a Trace MS (Finnigan Trace GC/MS, Finnigan, USA) equipped with a 3DB-624 Ultra Inert column (30 m × 250 μm × 1.4 μm, J&W Scientific, Folsom, CA, USA). The injector port was heated to 240 °C. The initial temperature was set to 38 °C for 5 min, then raised at 6 °C min^−1^ to 140 °C, elevated to 240 °C at 10 °C min^−1^ and held there for 10 min. The carrier gas was helium and had a flow rate of 1 mL min^−1^, and the split ratio was 1:10. Mass spectra were acquired in electron-impact mode. The MS was taken at 70 eV, and the ion-source temperature was 230 °C; the mass scanning range was 25–500 u. 

### 2.5. Statistical Analysis

Every experiment was performed in triplicate and results were expressed as the mean value ± standard deviation (SD) of three replicates. The SPSS 22.0 software (SPSS Inc., Chicago, IL, USA) was used for statistical data analysis. The statistical significance of the data was tested by one-way analysis of the variance (ANOVA), followed by the Duncan test to compare the means that showed significant variation (*p* < 0.05). 

For the qualitative and quantitative analyses of volatile substances: the obtained data were retrieved and identified with the NIST 2017 and Wiley 275 standard mass spectra. The volatile chemical components were identified, select positive and negative matching degrees greater than 85% of the substances were confirmed, and the peak-area-normalization method was used for qualitative and relative quantitative analyses. Bioinformatics software was used to draw the heat map and perform cluster analysis.

## 3. Results and Discussion

### 3.1. Effects of Different Cooking Methods on Nutrient Components in Different LEs

#### 3.1.1. Polysaccharide Content

**Figure 2:**
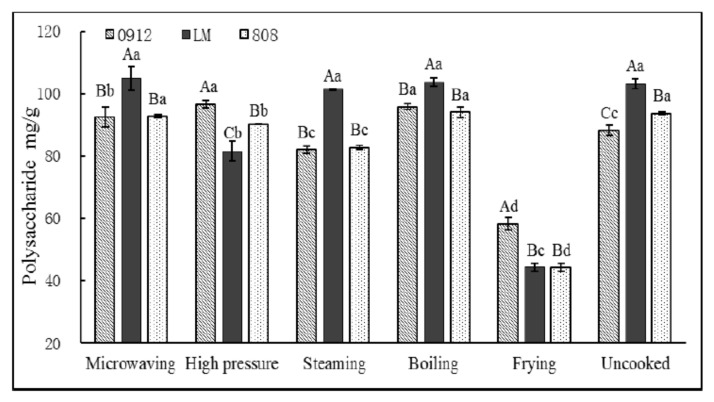
Effect of cooking on polysaccharide content in three kinds of *L. edodes.* A–C: significant difference (*p* < 0.05) in three kinds of *L. edodes* under the same treatment; a–d: significant difference (*p* < 0.05) in different processing methods. ANOVA and Duncan test were used to analyze the significant difference among samples from different cooking methods.

Polysaccharides are among the primary nutritional and bioactive components of LE and other mushrooms [27]. The effects of different cooking methods on the polysaccharide content of LE are shown in Figure 2. The polysaccharide content was the highest in LM, but the polysaccharide contents of the three varieties of LEs changed based on how they were processed. Microwaving and boiling could better retain the polysaccharides in LE, and the content of polysaccharides in 0912 was improved by 4.93% and 8.60%, respectively (*p* < 0.05). It shows that boiling and microwaving can destroy the cell structure of LE, especially for 0912, so more intracellular polysaccharides can be extracted. Fried LE showed the lowest polysaccharide content (*p* < 0.05); 0912, LM and 808 lost 34.1%, 57.1% and 52.8%, respectively. The reason may be that oil infiltrated into the structure of LE during frying, resulting in an increase in the mass ratio of fat in LE and a decrease in the mass ratio of polysaccharides. Another reason may be that the oil covered the surface of the cells, which hindered the dissolution and extraction of polysaccharides in the cells. There was a degradation of polysaccharides during frying, or the Maillard reaction, between polysaccharides and nitrogen-containing substances under a high-temperature environment [28], as we observed that the water in the LE evaporated rapidly and the color became darker in the frying process. Among them, different LE varieties showed different loss rates, which may be due to the differences in the composition and structure of polysaccharides in LE from different regions, resulting in differences in thermal stability, which needs to be further explored.

#### 3.1.2. Protein Content

**Figure 3:**
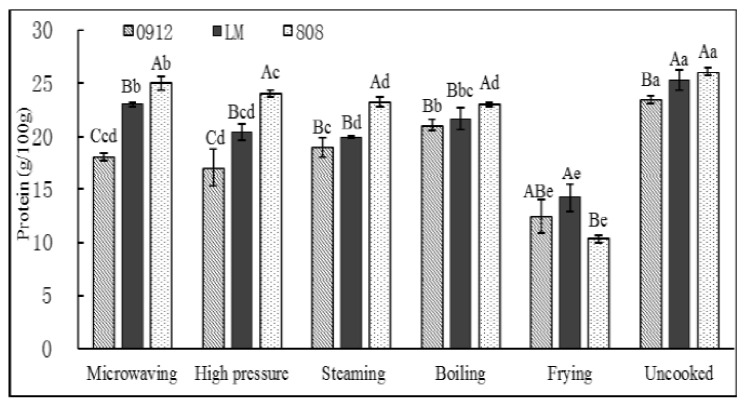
Effects of different cooking on protein content in three kinds of *L. edodes.* A–C: significant difference (*p* < 0.05) in three kinds of *L. edodes* under the same cooking methods; a–e: significant difference (*p* < 0.05) in different processing methods. ANOVA and Duncan test were used to analyze the significant difference among samples from different cooking methods.

The protein in LE is a high-quality source of plant protein [29]. It is well-known that heat treatment promotes protein denaturation or degradation, changing the quality of the protein composition of food [30]. As shown in Figure 3, it led to the decrease in protein content in LE. The critical effect of the cooking methods on protein was denaturation, which facilitated the digestion of the protein. However, the outcomes depended on the cooking conditions, with specific cooking methods (FR) more effectively inducing the denaturation of proteins than others, with loss rates of more than 44%. The higher lipid levels after frying may have induced a proportional dilution of the protein. This may be due to the destruction of the protein structure, degradation into primary and secondary structures or degradation to form polypeptides [30]. Erjavec et al. [31] and Nie et al. [9] reported that the protein in mushrooms was stable throughout thermal processes, and about 90% of the protein was water-insoluble, meaning that most of the protein remained in the mushroom after boiling, so the loss of protein (10~14%) was lower than FR (44~60%). However, the protein-loss rate of 808 was lower than that of 0912 and LM under other processing methods. Probably because of its own structure, 808 could tolerate higher temperature during cooking process than the others. Once again, 808 was the LE with major protein values compared with the other species.

#### 3.1.3. Dietary Fiber

**Figure 4:**
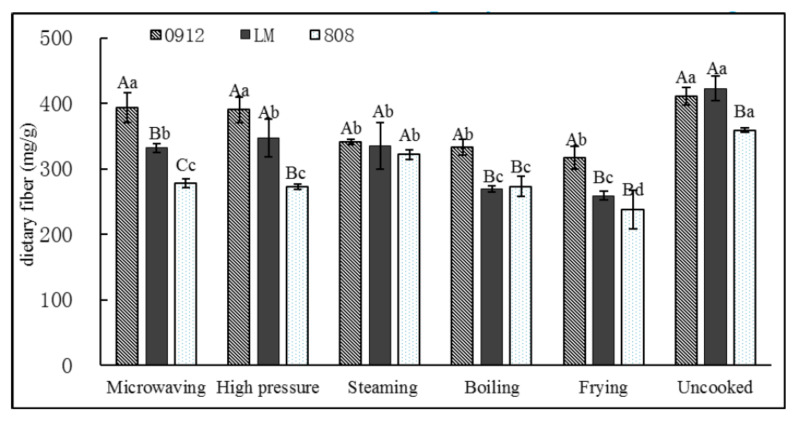
Effects of different processing methods on total dietary fiber content of *L. edodes*. A–C: significant difference (*p* < 0.05) in three kinds of *L. edodes* under the same cooking methods; a–d: significant difference (*p* < 0.05) in different processing methods. ANOVA and Duncan test were used to analyze the significant difference among samples from different cooking methods.

Edible fungi are good sources of dietary fiber (DF). DF plays an important role in maintaining the normal functions of the human body. As shown in Figure 4, the total dietary fiber (TDF) content of 0912 and LM without cooking treatment was about 42 mg/g, but 808 had a lower TDF (36 mg/g). TDF content was significantly reduced after boiling and frying compared to other groups, especially for LM, which had losses of 36% and 38%. This may be because the soluble dietary fiber of the LE was dissolved in the water and part of the cell-wall structure was destroyed during boiling/frying, resulting in the dense and orderly arrangement of the insoluble dietary fiber (IDF) being broken [32]. In addition, the loss rate of TDF of LM was also the largest in the MI (21.5%) and HP (17.8%) groups, which was very similar to that of polysaccharide. The decrease in TDF content was possibly mainly due to the IDF; the IDF in LE is mainly composed of cell-wall polysaccharides, and the contents of IDF and TDF in LM decreased rapidly in the initial stage of cooking. 

#### 3.1.4. Moisture Content

**Figure 5:**
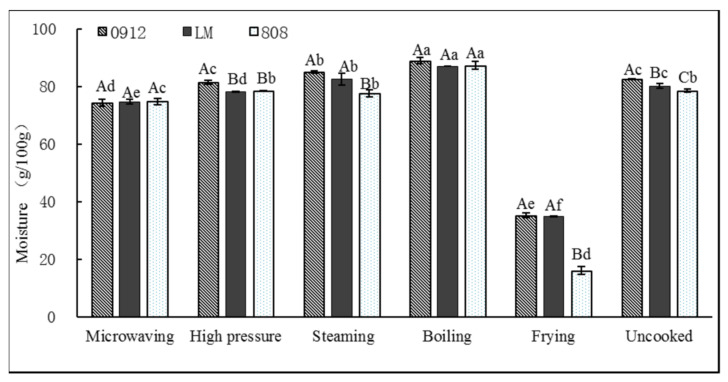
Effects of different processing methods on moisture content of *L. edodes.* A–C: significant difference (*p* < 0.05) in three kinds of *L. edodes* under same cooking; a–f: significant difference (*p* < 0.05) in different processing methods. ANOVA and Duncan test were used to analyze the significant difference among samples from different cooking methods.

As shown in Figure 5, the values for the moisture in the frying samples were found to be the lowest, with a reduction of up to 80%, while the content of water increased by 7.7~11.3% in LE after boiling. This may be due to the direct contact between LE and water during boiling, resulting in the filling of LE cells with water, while the water absorption and expansion of DF further increased the water content of the LE [22,33]. Similar results were observed in Ramırez-Anaya et al. [34]’s assay comparing raw and cooked vegetables: a strong reduction in moisture was detected in fried vegetables, while in samples cooked by other methods, only a slight decrease was noted. In the process of frying, the water in the sample evaporates rapidly and the oil enters the cells, reducing the water content. However, in LE with a low water content, the physical and chemical changes that occur during storage are slower, meaning it stores better. The LE did not directly come into contact with water under MI, HP and ST, so the water contents of the three kinds of LEs changed little. In addition to FR, the water contents of the three varieties of LEs processed were high, which is conducive to processing them into canned and soup products, while the water contents of the fried LEs were low, which is conducive to processing them into sauce products with high oil contents.

#### 3.1.5. Mineral Content

**Figure 6:**
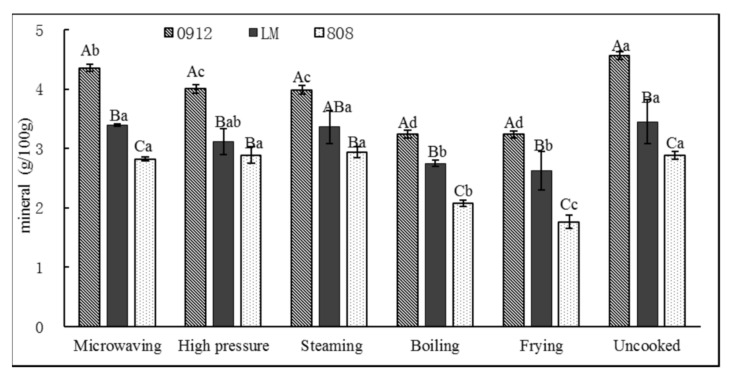
Effects of different processing methods on minerals content of *L. edodes.* A–C: significant difference (*p* < 0.05) in three kinds of *L. edodes* under the same cooking methods; a–c: significant difference (*p* < 0.05) in different processing methods. ANOVA and Duncan test were used to analyze the significant difference among samples from different cooking methods.

As shown in Figure 6, 0912 had the highest mineral content by 4.56 g/100 g, followed by LM (3.45 g/100 g) and 808 (2.88 g/100 g). The mineral contents of LM and 808 showed no significant differences from the control group after microwaving, steaming or high pressure (*p* > 0.05). Similarly, LE presented the highest values of moisture and less ash content. In the study, the mineral content decreased by 20.4~27.9% after boiling, and between 23.8% and 38.7% after frying, probably due to the leaching of soluble substances into the water or oil. Similar results were observed by Lee et al. [21] and Da Silva et al. [35]; P, Mg, K and other minerals of the LE decreased significantly in the process of boiling, which was mainly caused by leaching into the boiling water, while the reason for the decrease in the mineral content caused by frying may have been the increase in the proportion of fat, which resulted in an inevitable decrease in the mineral content. Comparing the types of LEs, 0912 had the major ash content values, followed by LM and 808. Nonetheless, only one cooking condition (i.e., cooking time and temperature combination) was employed for each cooking method in this study, and hence the nutrient components of LE at different cooking times and temperature combinations were not completely reflected.

#### 3.1.6. Total Phenolic Content

**Figure 7:**
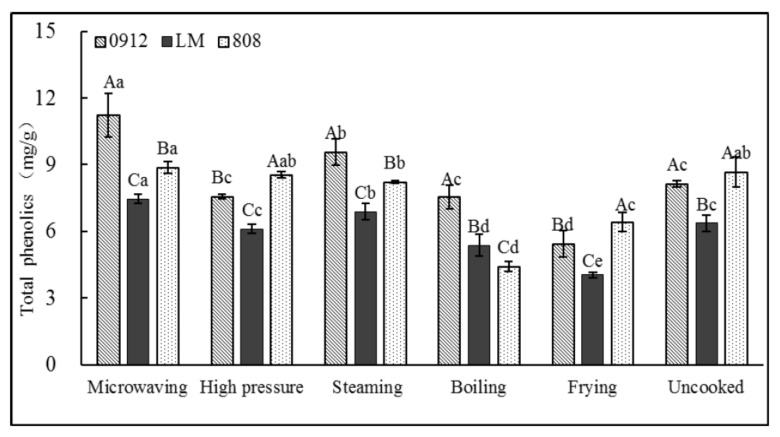
Effects of different processing methods on total phenolics of *L. edodes.* A–C: significant difference (*p* < 0.05) in three kinds of *L. edodes* under the same cooking methods; a–e: significant difference (*p* < 0.05) in different processing methods. ANOVA and Duncan test were used to analyze the significant difference among samples from different cooking methods.

LE is rich in phenolic compounds and has good bioactive functions, but cooking significantly affects the retention and availability of the phenolic compounds [36]. As shown in Figure 7, the TP content in the unprocessed LE was 6.37~8.68 mg/g. The LE sample had the highest TP after microwaving and had the lowest after frying and boiling, which may be due to the direct contact between the LE and water or oil resulting in the loss of phenolic substances into the heating medium. The loss of the TP content of LE was 26~36% after frying, followed by boiling. Similar observations in the potato have been reported by Tian et al. [37]. On the one hand, it may be due to the frying temperature being the highest, thereby inducing greater damage to the phenolic substances. It has been established that polyphenols are sensitive to heat and pressure [12,38], so the results obtained here could have been influenced by the cooking methods. On the other hand, it is possible that fat-soluble phenols were dissolved in the oil or participated in the oxidation reaction of the oil. It is possible that 808 contains more hydrophilic phenolic substances, which leads to dissolution and release from LE into boiling water, resulting in a high loss rate. This suggests that the heating duration and presence of a direct cooking medium (e.g., water) are likely to be the determining factors that influenced the TP of the LE. The presence of a hydroxyl group (–OH) in phenolic compounds might enhance their ability to form hydrogen-bonded clusters with water molecules that cause the leaching of phenolic acids into the surrounding cooking water. 

By contrast, in HP, MI and ST, the mushroom block was not in direct contact with the heating medium, meaning that the phenolic substances could be better retained. We found that the polyphenol content of the LE was significantly increased after steaming or microwaving, compared to other cooking methods, by 8~18% and 17~38%, respectively. Previous studies also reported an increase in the polyphenol content and the antioxidant activity of microwaved *L. edodes* [22]. It may be that heat treatment softens the tissue of LE, thus improving the yield of phenolic compounds extracted from the cell matrix. In addition, due to the cleavage of glycosidic bonds, a high temperature increases the release of phenolic acids from conjugated glycosides, so it reacts better with the Folin reagent in phenol determination [39]. In general, microwaving and steaming can maintain and improve the total phenol content in LE.

### 3.2. Effects of Different Processing Methods on Antioxidant Activity of Lentinus edodes

**Figure 8:**
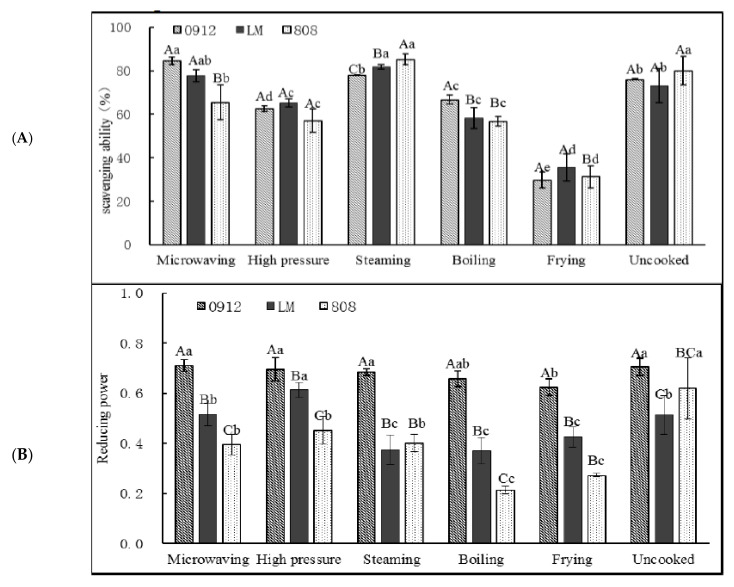
Effects of different processing methods on antioxidant activity of *Lentinus edodes*. (**A**): DPPH-scavenging activity in three kinds of *Lentinula edodes* with different cooking. DPPH-radical-scavenging effect of Vc: 95.23% ± 1.44% with 1 mg/mL. (**B**): Reducing power in three kinds of Lentinula edodes with different cooking methods. Reducing power of Vc: 1.09 ± 0.04 with 1 mg/mL. All data are the average of three experiments. A–C: significant difference (*p* < 0.05) in three kinds of *L. edodes* under the same cooking methods; a–c: significant difference (*p* < 0.05) in different processing methods. ANOVA and Duncan test were used to analyze the significant difference among samples from different cooking methods.

Antioxidants play a major role in preventing free-radical formation and harmful activities that damage DNA, lipids, proteins and other biomolecules. It should be noted that each antioxidant test is based on different principles and mechanisms [40], so it is possible for a food sample to show high antioxidant activity according to one measuring method but not according to another [41]. Therefore, different antioxidant assays should be used to measure antioxidant activity. LE contains polyphenols, polysaccharides and other antioxidant components, which have good antioxidant activity [10]. As shown in Figure 8, the three kinds of LEs showed good scavenging abilities for DPPH free radicals after microwaving and steaming, ranging from 73~76% to 78~85%, which may indicate that the polysaccharides and phenols in the LE were well-retained. A decrease in antioxidant activity in the boiled (57~67%) and fried (30~36%) samples was also detected (*p* < 0.05). As shown in Figure 8, MI and HP could better retain the reducing power of LE, while BO and FR weakened the reduction ability of LE, especially for LM and 808 (*p* < 0.05). Previously, several authors demonstrated that the boiling process significantly decreases the antioxidant activities and polyphenol contents in different mushroom varieties [41,42]. Furthermore, various antioxidant substances are leached into the water during boiling, which results in a decrease in the food’s antioxidant activities [43]. The finding from this study showed a similar trend to that reported by Choi et al. [44] in Shiitake mushroom and Chang et al. [14] in *Hericium erinaceus*. The reducing power differed considerably between the three kinds of uncooked LEs, probably due to the particular structure and shape of each mushroom variety, which affected the antioxidant material that was finally released from the mushroom [45]. Then, the reducing power of the three kinds of LEs changed differently during cooking. For example, the reducing power of 0912 decreased only in FR (*p* < 0.05); the reduction ability of LM was improved in HP (*p* < 0.05) and could be well-retained in MI (*p* > 0.05); the reducing power of 808 was decreased significantly after all kinds of processing (*p* < 0.05). Overall, the reducing power of 0912 (0.62~0.71) was greater than that of LM (0.37~0.61) and 808 (0.27~0.45) (*p* < 0.05), which may be related to the retention of polysaccharides, polyphenols, functional proteins and other antioxidant compounds in LE. 

According to these results, various antioxidant substances are leached into the water during boiling, which decreases the food’s antioxidant capacities. Microwaving seems to be one of the best cooking processes for preserving the antioxidant properties of mushrooms, as demonstrated by Tan et al. [13] and Irene et al. [22]. During cooking, enzymes induce numerous physical and chemical reactions, such as the Maillard reaction, Strecker degradation and the hydrolysis of esters and glycosides, which leads to the generation of new antioxidant compounds [44]. As stated above, the increase could be explained by the release of antioxidant compounds that were previously bound to other molecules, thereby increasing the polyphenol content and antioxidant activity during thermal treatment [22,45]. The antioxidant activity of mushroom polysaccharides has been widely reported in recent years, and we speculate that the dissolution and digestion of polysaccharides and polyphenols might be among the reasons for this antioxidant activity, which needs further research to prove.

### 3.3. Effects of Different Cooking Methods on Nonvolatile Taste-Active Substances of Lentinus edodes

#### 3.3.1. Free Amino Acids

Numerous studies have reported that mushrooms are a potential source of essential amino acids (EAAs). The effects of different cooking methods on the free amino acid (FAA) compositions in the three kinds of LEs (17 amino acids were detected) are shown in Table 1. The total FAA and EAA contents in 0912 and 808 were higher than those in LM, but their contents decreased with processing. These losses were probably due to the Strecker degradation of amino acids or the Maillard reaction between amino acids and reducing sugars during cooking. Boiling and frying most significantly reduced the FAA content (*p* < 0.05), followed by pressure cooking. However, HP and FR increased the proportions of sweet and umami amino acids and decreased the proportion of bitter amino acids (Table 2), especially for 0912 and LM. We suggest that this may positively affect the taste of the LE product. Most of the FAA was easily dissolved in hot water, so its content in mushroom broth tends to increase [9,46]. Therefore, boiling only reduced the FAA; it did not increase the proportions of umami or sweet amino acids.

The levels of some amino acids, such as glutamate, isoleucine, lysine and glycine in 0912 (*p* < 0.05) and serine, methionine and isoleucine in 808 (*p* < 0.05), increased after microwaving. There is a possibility that FAAs are generated by the induction of favorable changes in the protein structure under various cooking methods, but higher temperatures could result in unfavorable structural changes that reduce the susceptibility of proteins to enzymatic hydrolysis [47,48]. Among the amino acids, aspartate, glutamate, glycine, alanine and arginine are responsible for a palatable taste [6]. In this study, the glutamate content seemed to be the highest of the 17 amino acids detected in LM and 808, followed by alanine and valine, and alanine and arginine, respectively. The methionine content seemed to be the highest in 0912. Glutamate and alanine are umami and sweet amino acids, respectively, while methionine is a bitter amino acid, which may make LM and 808 taste better than 0912, especially as 808 had the highest proportion of glutamate by 2.2~5.1 mg/g. There are synergistic effects among amino acids. For example, the umami of LE can be improved when aspartate and glutamate combine. 

#### 3.3.2. Nucleotide Levels

The unique taste of LE is not only related to the rich FAAs but also closely related to the content of 5′-nucleotides, which are flavor enhancers [49]. These include 5′-IMP, the major taste-active component in mushrooms [18]. The nucleotide levels in mushrooms after different cooking methods are listed in Table 3. While 808 is rich in three nucleotides, it was determined that the five cooking methods all destroyed the nucleotides in mushrooms. The loss rates for nucleotides caused by frying and boiling were higher than those for the other three cooking methods—65~78% and 59~72%, respectively—while the loss rate for microwave treatment was the lowest, which might be attributed to the short cooking time of microwaving. This may be explained by several factors: first, microwaving is gentle, so it does not damage nucleotide functionality, and second, nucleotide deaminase retains its enzymatic activity after microwaving. The content of 5′-AMP, which has a sweet flavor, was higher than the contents of 5′-IMP and 5′-CMP, indicating that 5′-AMP has good heat resistance. In addition, the loss rates for the three nucleotides in 808 were lower than those in 0912 and LM under the same processing conditions, which indicates that 808 has good thermal stability. When flavor amino acids and flavor nucleotides are present together, the synergistic effect of the two improves the flavor intensity of LE. We also found that 808 had higher contents of amino acids and nucleotides, suggesting that perhaps diced 808 mushrooms tasted better after processing.

### 3.4. Effects of Different Cooking Methods on Volatile Compounds of Lentinus edodes

**Figure 9:**
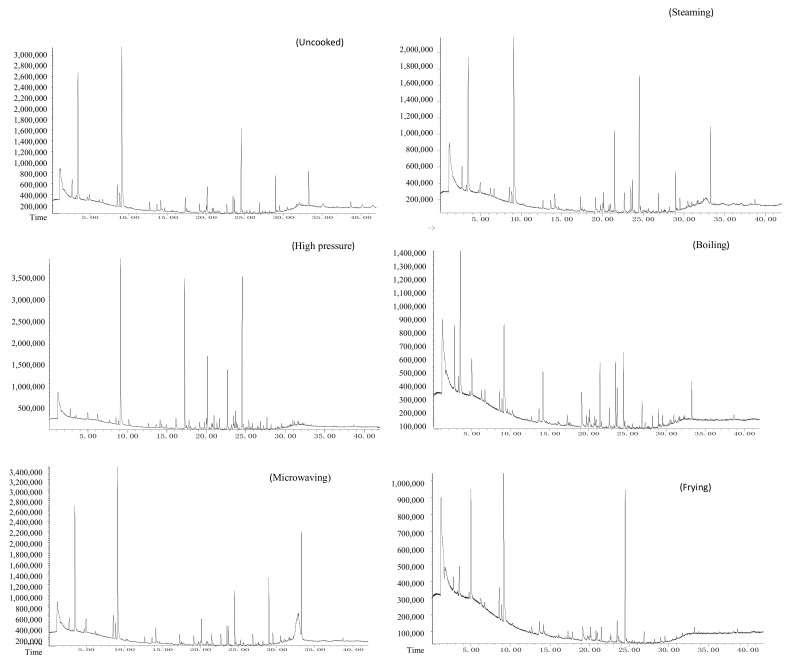
Gas chromatography–mass spectrometry of LE from different cooking methods.

**Figure 10:**
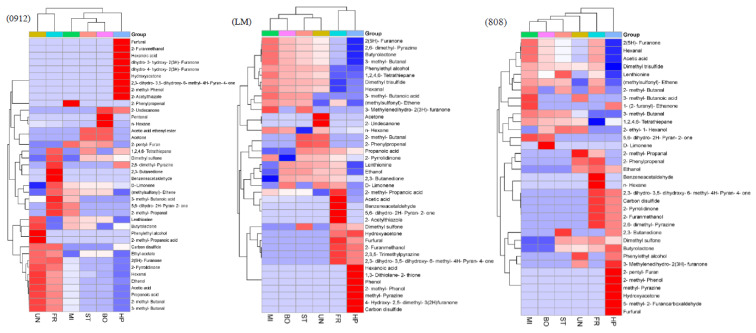
Heat map of volatile flavor compounds in different cooking treatments in three kinds of *L. edodes*. ST, steam treatment; BO, boil treatment; FR: fry treatment, MI: microwave treatment; HP: high-pressure treatment; UN: uncooked.

In food processing and preparation, volatile flavor compounds are often generated through the Maillard reaction, lipid oxidation and degradation, caramelization reactions, etc. These aromatic compounds can enrich and enhance the food flavor system, so they have great significance in food research. The heat map is an intuitive and visual method for analyzing the distribution of experimental data. It can cluster data and samples to determine the quality of the samples [50]. Figure 9 and Figure 10 showed the types of volatile flavor compounds detected in different kinds of LEs after cooking via HS-SPME–GC/MS. The volatile compounds are representative, demonstrating the presence of 16, 21 and 15 compounds in uncooked 0912, LM and 808 mushrooms, respectively. This shows that flavor compounds are abundant in raw LM. The identified aroma compounds include alcohols, aldehydes, ketones, acids, esters, sulfur compounds and pyrazines. There were more volatile compounds after pressure cooking than in the uncooked LE because of the longer cooking time, followed by frying, while there were fewer volatile compounds in the LM and 808 following boiling and microwaving than for the uncooked samples. *Lentinus edodes* mainly produce aldehydes, ketones, pyrazines, esters and sulfur-containing compounds after cooking. These flavor substances together affect the final sensory quality of the products, but their contribution to food flavors varies with the substance threshold [18]. Ketones have a unique fragrance and fruity flavor; those we detected included 2(5H)-furanone, 2-pyrrolidone and 2,3-butanedione. The aldehydes detected included 2-methylbutyraldehyde, hexanal and 3-methylbutyraldehyde. They are the most typical volatile compounds, which are mainly produced by the oxidation of polyunsaturated fatty acids, and the threshold is very low, which has a certain effect on the distribution of mushroom products. Alcohols have mild plant aromas; they are considered to contribute little to the overall flavors of mushroom products due to their high thresholds. The esters detected included 4-hydroxybutyrolactone. Some heterocyclic compounds formed in the Maillard reaction were important for the special flavor profiles of the three kinds of LEs, including substituted furans, pyrroles and pyrazines. The greater the number of volatile substances detected, the higher the degree of protein hydrolysis in the sample, the more likely the FAAs produced from protein hydrolysis were to participate in the Maillard reaction, and the greater the number of flavor substances that were formed in the reaction [14,51].

The relative contents of the compounds in mushrooms subject to different cooking methods are shown in Table 4. The quantity and content of ethanol in the LE were decreased significantly compared to those in the control group, especially for 0912 and 808. This may be attributed to evaporation during the high-temperature cooking, but furfuryl alcohol was only found following pressure cooking and frying; 2-ethyl-1-hexanol was only found in 808; they were not mentioned in previous mushroom products. During the cooking process, the compositions of aroma compounds changed due to the partial loss of the existing compounds and the formation of new ones as a result of various chemical reactions. While some ketones’ contents increased after high-pressure cooking or frying, such as hydroxyacetone and 2,3-dihydro-3,5-dihydroxy-6-methyl-4h-pyran-4-one, which was due to the degradation of polyunsaturated fatty acids. The fried samples contained higher aldehyde contents than the other groups for 0912 and 808, especially for phenylacetaldehyde, which could be produced by the Strecker degradation of phenylalanine in the Maillard reaction [52]. Accordingly, furfural only appeared in high-pressure cooking or frying, and 5-methyl-2-furancarboxaldehyde was only present in the high-pressure processing of 808. They may be derived from hydroperoxide and carbohydrate degradation products, but they were not reported in *A. bisporus* L. or oyster mushrooms [18]. The content of pyrazine increased after frying or high-pressure cooking, which may have occurred due to the thermal degradation of threonine and serine. It is reported that alkyl pyrazine is formed in the Maillard reaction in the presence of alanine, glycine, valine, isoleucine and leucine [53], and the contents of these amino acids in LE were decreased significantly after FR (Table 1). Many volatile compounds have been reported to be the major aroma compounds for mushrooms, such as sulfur compounds. These still made up a large portion of the aroma compounds of the three kinds of LEs after cooking, especially steaming. It was also found that 808 and LM had higher proportions of sulfur compounds in different LE species. Lentinan and 1,2,4,6-tetrathione were the major sulfur compounds, accounting for more than 35% of the total volatile compounds in *Lentinus edodes*. In addition, (methylsulfonyl)-ethene was newly generated after processing, except for high-pressure cooking. The proportions of acids all decreased after processing; there was no propanoic acid, 2-methyl-propanoic acid or hexanoic acid in 808, and hexanoic acid only appeared in 0912 and LM under HP. The 0912 and 808 varieties contained a higher ester content after HP than in the other groups. For the esters and acids, although their contents did not account for very high proportions in LE, they play important roles in the overall aroma profile. 

The results indicate that the cooking method is an important factor impacting the formation of volatile compounds through the Maillard reaction and lipid oxidation, and an increase in the cooking temperature increases the formation of these flavor compounds. However, the mechanisms and pathways behind the flavors produced with different cooking techniques are poorly understood and should be further studied to better clarify the effects of cooking techniques on the generation of aromatic compounds.

## 4. Conclusions

In this paper, five different cooking methods (steaming, boiling, pressure cooking, frying and microwaving) were used to cook three varieties of *Lentinus edodes* (808, 0912 and LM) from Guizhou Province. The polysaccharides were the most abundant in LM, and the minerals were abundant in 0912, while LM, 808 and 0912 had low amounts of polyphenols, dietary fiber and proteins, respectively. We found that the cooking method had a great influence on the nutrient contents, antioxidant activities and flavor compounds in *Lentinus edodes*. Overall, it is difficult for any cooking method to retain all nutrients at the highest level. The dietary fiber and protein contents were decreased during cooking. Microwaving was the best method for retaining high levels of polysaccharides and polyphenols, which are needed for the mushrooms to exhibit strong antioxidant activity. Each cooking method produced a unique flavor. The frying and high-pressure treatments caused severe losses of FAAs and nucleotides, but they increased the proportions of sweet and umami amino acids and volatile compounds. HS-SPME–GC/MS detected 16, 21 and 15 volatile substances in the three varieties of *Lentinus edodes*, which changed significantly under different processing conditions. The concentrations of some volatile components were reduced after cooking, such as alcohols and acids, while some volatile components were enhanced after pressure cooking. Additionally, the sulfur compounds were increased after microwaving and steaming. It seemed that microwaving was an excellent choice for cooking LE to support nutrient retention and antioxidant activity. Nevertheless, most of the volatile compounds’ concentrations were increased most significantly after pressure cooking, followed by frying. In terms of different varieties of *Lentinus edodes*, 0912 can better retain nutrients, while 808 has better flavor characteristics after processing, and LM has weak thermal processing stability. In summary, these findings suggest that each method has a characteristic effect on *Lentinus edodes*’ properties. The next project will further explore the molecular mechanism underlying the formation of flavor compounds in LE.

## Figures and Tables

**Table 1:** Effect of cooking methods on free amino acids in three kinds of *L. edodes* (mg/g).

Amino Acids	0912						LM						808					
	UN	ST	HP	MI	BO	FR	UN	ST	HP	MI	BO	FR	UN	ST	HP	MI	BO	FR
Asp	0.97 ± 0.00 ^a^	0.77 ± 0.24 ^ab^	1.09 ± 0.03 ^a^	0.98 ± 0.07 ^a^	0.49 ± 0.21 ^b^	0.57 ± 0.06 ^b^	0.28 ± 0.03 ^b^	0.21 ± 0.01 ^b^	0.67 ± 0.02 ^a^	0.25 ± 0.00 ^b^	0.27 ± 0.01 ^b^	0.28 ± 0.21 ^b^	1.02 ± 0.01 ^a^	0.95 ± 0.01 ^a^	0.67 ± 0.06 ^ab^	0.53 ± 0.2 ^b^	0.43 ± 0.16 ^b^	0.41 ± 0.16 ^b^
Glu	1.75 ± 0.02 ^b^	1.68 ± 0.08 ^b^	1.78 ± 0.01 ^b^	2.02 ± 0.06 ^a^	1.08 ± 0.02 ^c^	1.17 ± 0.09 ^c^	2.69 ± 0.07 ^a^	2.16 ± 0.03 ^d^	2.02 ± 0.05 ^c^	2.38 ± 0.01 ^b^	1.24 ± 0.03^e^	1.30 ± 0.09^e^	5.12 ± 0.21 ^a^	4.26 ± 0.15 ^b^	3.52 ± 0.01 ^c^	4.40 ± 0.0 ^b^	2.34 ± 0.20 ^d^	2.20 ± 0.03 ^d^
Ser	0.30 ± 0.01 ^a^	0.34 ± 0.04 ^a^	0.34 ± 0.03 ^a^	0.31 ± 0.00 ^a^	0.17 ± 0.09 ^b^	0.06 ± 0.00 ^c^	0.17 ± 0.00 ^a^	0.16 ± 0.02 ^b^	0.14 ± 0.01 ^a^	0.15 ± 0.01 ^ab^	0.08 ± 0.01 ^c^	0.06 ± 0.01 ^c^	0.14 ± 0.09 ^b^	0.28 ± 0.06 ^a^	0.08 ± 0.06 ^b^	0.27 ± 0.0 ^a^	0.05 ± 0.04 ^b^	0.05 ± 0.03 ^b^
Gly	0.62 ± 0.00 ^c^	0.59 ± 0.02 ^c^	0.82 ± 0.01 ^a^	0.70 ± 0.04 ^b^	0.34 ± 0.01 ^d^	0.38 ± 0.02 ^d^	0.46 ± 0.03 ^a^	0.42 ± 0.01 ^a^	0.49 ± 0.02 ^b^	0.40 ± 0.00 ^b^	0.22 ± 0.01 ^d^	0.28 ± 0.02 ^c^	0.64 ± 0.05 ^a^	0.57 ± 0.02 ^ab^	0.52 ± 0.01 ^b^	0.53 ± 0.00 ^b^	0.27 ± 0.01 ^c^	0.34 ± 0.05 ^c^
Thr	1.36 ± 0.01 ^a^	1.31 ± 0.06 ^a^	1.35 ± 0.04 ^a^	1.22 ± 0.00 ^b^	0.89 ± 0.02 ^c^	0.86 ± 0.00 ^c^	0.95 ± 0.07 ^a^	0.91 ± 0.02 ^a^	0.95 ± 0.02 ^a^	0.89 ± 0.01 ^a^	0.51 ± 0.01 ^b^	0.56 ± 0.02 ^b^	1.28 ± 0.14 ^a^	1.09 ± 0.01 ^b^	1.01 ± 0.00 ^b^	1.07 ± 0.05 ^b^	0.60 ± 0.05 ^c^	0.65 ± 0.08 ^c^
Ala	2.08 ± 0.02 ^a^	2.03 ± 0.06 ^ab^	1.97 ± 0.01 ^b^	1.85 ± 0.01 ^c^	1.22 ± 0.02 ^d^	1.30 ± 0.02 ^d^	1.33 ± 0.05 ^a^	1.21 ± 0.02 ^b^	1.24 ± 0.01 ^b^	1.23 ± 0.02 ^b^	0.63 ± 0.01 ^d^	0.76 ± 0.04 ^c^	2.05 ± 0.15 ^a^	1.77 ± 0.00 ^b^	1.65 ± 0.02 ^b^	1.77 ± 0.06 ^b^	0.90 ± 0.03 ^c^	1.02 ± 0.04 ^c^
Pro	0.52 ± 0.04 ^a^	0.58 ± 0.02 ^a^	0.73 ± 0.12 ^a^	0.56 ± 0.01 ^a^	0.43 ± 0.21 ^a^	0.45 ± 0.15 ^a^	0.74 ± 0.14 ^a^	0.79 ± 0.13 ^a^	0.68 ± 0.14 ^ab^	0.72 ± 0.06 ^a^	0.43 ± 0.04 ^c^	0.55 ± 0.07 ^ab^	0.84 ± 0.21 ^a^	0.76 ± 0.05 ^a^	0.28 ± 0.03 ^c^	0.68 ± 0.02 ^ab^	0.47 ± 0.14 ^abc^	0.36 ± 0.27 ^bc^
His	0.35 ± 0.00 ^a^	0.45 ± 0.12 ^a^	0.33 ± 0.01 ^a^	0.44 ± 0.19 ^a^	0.39 ± 0.48 ^a^	0.12 ± 0.11 ^a^	0.13 ± 0.02 ^b^	0.25 ± 0.03 ^a^	0.06 ± 0.02 ^c^	0.14 ± 0.02 ^b^	0.14 ± 0.00 ^b^	0.08 ± 0.01 ^c^	1.10 ± 1.21 ^a^	0.10 ± 0.01 ^a^	0.20 ± 0.09 ^a^	0.17 ± 0.04 ^a^	0.09 ± 0.00 ^a^	0.13 ± 0.02 ^a^
Arg	1.22 ± 0.02 ^a^	1.06 ± 0.04 ^b^	1.13 ± 0.03 ^b^	1.21 ± 0.04 ^a^	0.84 ± 0.01 ^c^	0.80 ± 0.04 ^c^	0.83 ± 0.03 ^a^	0.58 ± 0.05 ^c^	0.50 ± 0.01 ^c d^	0.72 ± 0.03 ^b^	0.41 ± 0.09 ^d^	0.42 ± 0.02 ^d^	1.83 ± 0.08 ^a^	1.49 ± 0.04 ^c^	1.30 ± 0.01 ^d^	1.61 ± 0.00 ^b^	0.96 ± 0.04^e^	0.88 ± 0.01^e^
Val	1.67 ± 0.04 ^a^	1.61 ± 0.11 ^a^	1.75 ± 0.01 ^a^	1.65 ± 0.08 ^a^	0.79 ± 0.02 ^b^	0.78 ± 0.03 ^b^	1.32 ± 0.00 ^a^	1.26 ± 0.08 ^ab^	1.29 ± 0.03 ^ab^	1.19 ± 0.04 ^b^	0.69 ± 0.05 ^c^	0.68 ± 0.01 ^c^	1.61 ± 0.01 ^a^	1.51 ± 0.00 ^a^	1.13 ± 0.03 ^b^	1.58 ± 0.07 ^a^	0.68 ± 0.04 ^c^	0.71 ± 0.04 ^c^
Met	4.00 ± 0.03 ^a^	3.71 ± 0.11 ^a^	1.56 ± 0.06 ^d^	2.99 ± 0.10 ^b^	3.08 ± 0.12 ^b^	2.45 ± 0.26 ^c^	1.25 ± 0.09 ^a^	1.22 ± 0.14 ^a^	0.48 ± 0.09 ^b^	1.28 ± 0.03 ^a^	0.70 ± 0.13 ^b^	0.48 ± 0.29 ^b^	0.47 ± 0.14 ^b^	0.83 ± 0.02 ^a^	0.26 ± 0.01 ^bc^	0.99 ± 0.04 ^a^	0.19 ± 0.21 ^c^	0.22 ± 0.04 ^bc^
Phe	0.99 ± 0.08 ^a^	0.89 ± 0.08 ^a^	1.00 ± 0.01 ^a^	1.05 ± 0.17 ^a^	0.50 ± 0.01 ^b^	0.50 ± 0.03 ^b^	0.87 ± 0.05 ^a^	0.71 ± 0.00 ^a^	0.67 ± 0.01 ^a^	0.75 ± 0.06 ^a^	0.36 ± 0.19 ^b^	0.38 ± 0.07 ^b^	1.02 ± 0.02 ^a^	0.71 ± 0.02 ^bc^	0.81 ± 0.15 ^b^	0.72 ± 0.01 ^bc^	0.59 ± 0.07 ^c^	0.54 ± 0.08 ^c^
Ile	0.72 ± 0.01 ^b^	0.65 ± 0.01 ^c^	0.89 ± 0.01 ^a^	0.85 ± 0.04 ^a^	0.36 ± 0.00 ^d^	0.39 ± 0.00 ^d^	0.51 ± 0.02 ^ab^	0.54 ± 0.03 ^a^	0.39 ± 0.01 ^abc^	0.45 ± 0.06 ^abc^	0.32 ± 0.07 ^c^	0.34 ± 0.15 ^bc^	1.07 ± 0.08 ^b^	1.18 ± 0.05 ^a^	0.69 ± 0.00 ^c^	1.21 ± 0.01 ^a^	0.47 ± 0.01 ^d^	0.50 ± 0.01 ^d^
Leu	1.13 ± 0.01 ^b^	0.96 ± 0.04 ^c^	1.29 ± 0.01 ^a^	1.27 ± 0.03 ^ab^	0.73 ± 0.05 ^d^	0.75 ± 0.13 ^d^	0.89 ± 0.00 ^a^	0.71 ± 0.01 ^b^	0.67 ± 0.01 ^b^	0.71 ± 0.01 ^b^	0.45 ± 0.11 ^c^	0.49 ± 0.03 ^c^	1.38 ± 0.77 ^a^	1.33 ± 0.01 ^a^	1.25 ± 0.06 ^a^	1.38 ± 0.05 ^a^	0.75 ± 0.00 ^a^	0.84 ± 0.00 ^a^
Tyr	1.41 ± 0.01 ^a^	1.30 ± 0.06 ^a^	1.28 ± 0.01 ^a^	1.25 ± 0.04 ^a^	0.69 ± 0.18 ^b^	0.61 ± 0.06 ^b^	0.43 ± 0.01 ^a^	0.41 ± 0.03 ^a^	0.40 ± 0.06 ^a^	0.44 ± 0.38 ^a^	0.36 ± 0.07 ^a^	0.24 ± 0.05 ^a^	0.05 ± 0.02 ^a^	0.04 ± 0.00 ^a^	0.03 ± 0.00 ^a^	0.11 ± 0.12 ^a^	0.03 ± 0.01 ^a^	0.07 ± 0.07 ^a^
Cys	0.46 ± 0.04 ^a^	0.16 ± 0.08 ^b^	0.21 ± 0.03 ^b^	0.24 ± 0.05 ^ab^	0.21 ± 0.20 ^b^	0.25 ± 0.00 ^ab^	0.09 ± 0.09 ^a^	0.03 ± 0.00 ^a^	0.03 ± 0.00 ^a^	0.03 ± 0.02 ^a^	0.04 ± 0.01 ^a^	0.03 ± 0.01 ^a^	0.50 ± 0.00 ^abc^	0.68 ± 0.13 ^a^	0.48 ± 0.06 ^abc^	0.54 ± 0.08 ^ab^	0.44 ± 0.08 ^bc^	0.32 ± 0.05 ^c^
Lys	0.03 ± 0.01 ^b^	0.03 ± 0.01 ^b^	0.03 ± 0.01 ^b^	0.07 ± 0.02 ^a^	0.02 ± 0.00 ^b^	0.03 ± 0.01 ^b^	1.04 ± 0.02 ^a^	0.95 ± 0.01 ^b^	0.87 ± 0.01 ^c^	0.95 ± 0.04 ^b^	0.59 ± 0.01 ^d^	0.57 ± 0.04 ^d^	0.99 ± 0.18 ^a^	1.08 ± 0.02 ^a^	0.58 ± 0.80 ^a^	1.06 ± 0.04 ^a^	0.27 ± 0.35 ^a^	0.19 ± 0.09 ^a^
EAA	11.27 ± 0.19 ^a^	10.42 ± 0.46 ^b^	9.11 ± 0.16 ^c^	10.28 ± 0.45 ^b^	7.04 ± 0.39 ^d^	6.35 ± 0.56 ^d^	6.84 ± 0.25 ^a^	6.29 ± 0.30 ^b^	5.32 ± 0.18 ^c^	6.22 ± 0.25 ^b^	3.61 ± 0.57 ^d^	3.51 ± 0.62 ^d^	7.83 ± 1.34 ^a^	7.73 ± 0.13 ^a^	5.73 ± 1.04 ^b^	8.02 ± 0.25 ^a^	3.54 ± 0.74 ^c^	3.65 ± 0.34 ^c^
Total	19.55 ± 0.16 ^a^	18.07 ± 0.75 ^b^	17.52 ± 0.13 ^b^	18.64 ± 0.45 ^ab^	12.25 ± 0.88 ^c^	11.47 ± 0.62 ^c^	14.00 ± 0.71 ^a^	12.50 ± 0.63 ^b^	11.56 ± 0.53 ^c^	12.69 ± 0.79 ^b^	7.44 ± 0.85 ^d^	7.52 ± 1.14 ^d^	21.10 ± 2.99 ^a^	18.62 ± 0.10 ^a^	14.44 ± 0.78 ^b^	18.64 ± 0.38 ^a^	9.52 ± 0.03 ^c^	9.43 ± 0.67 ^c^

Each value is expressed as the mean ± SD (n = 3) of triplicate determinations. ANOVA and Duncan test were used to analyze the significant difference among samples from different cooking methods. Means with different letters within a row were significantly different (*p* < 0.05) in different cooking methods. EAA, essential amino acid; Val, valine; Thr, threonine; Met, methionine; Leu, leucine; Ile, isoleucine; Trp, tryptophan; Phe, phenylalanine; Lys, lysine; Gly, glycine; His, histidine; Tyr, tyrosine; Asp, aspartic acid; Ser, serine; Cys, cysteine; Arg, arginine; Ala, alanine; Glu, glutamate. ST, steam treatment; BO, boil treatment; FR: fry treatment, MI: microwave treatment; HP: high-pressure treatment; UN: uncooked.

**Table 2:** The content and proportion of flavoring amino acids of *L. edodes* produced in Guizhou after different processing methods.

Varieties	Flavor Characteristics	UN		ST		HP		MI		BO		FR	
		Content (mg/g)	Proportion/%	Content (mg/g)	Proportion/%	Content (mg/g)	Proportion/%	Content (mg/g)	Proportion/%	Content (mg/g)	Proportion/%	Content (mg/g)	Proportion/%
0912	umami	2.72 ± 0.02 ^ab^	13.91	2.45 ± 0.33 ^b^	13.41	2.87 ± 0.04 ^ab^	16.38	3.00 ± 0.13 ^a^	16.09	1.58 ± 0.23 ^c^	12.90	1.74 ± 0.15 ^c^	15.17
	sweet	4.87 ± 0.08 ^ab^	24.96	4.83 ± 0.20 ^ab^	26.73	5.19 ± 0.20 ^a^	29.62	4.64 ± 0.07 ^b^	24.89	3.06 ± 0.36 ^c^	24.98	3.05 ± 0.19 ^c^	26.59
	bitter	10.07 ± 0.20 ^a^	51.51	9.31 ± 0.50 ^a^	51.52	7.94 ± 0.16 ^b^	45.32	9.46 ± 0.65 ^a^	50.75	6.68 ± 0.70 ^c^	54.53	5.79 ± 0.60 ^d^	50.48
	tasteless	1.89 ± 0.06 ^a^	9.67	1.49 ± 0.15 ^a^	8.25	1.52 ± 0.05 ^a^	8.68	1.55 ± 0.11 ^a^	8.32	0.93 ± 0.38 ^b^	7.59	0.89 ± 0.07 ^b^	7.76
LM	umami	2.97 ± 0.10 ^a^	21.21	2.37 ± 0.04 ^c^	18.96	2.69 ± 0.08 ^b^	23.27	2.64 ± 0.01 ^b^	20.80	1.51 ± 0.04 ^d^	20.30	1.59 ± 0.31 ^d^	21.14
	sweet	3.67 ± 0.29 ^a^	26.21	3.48 ± 0.20 ^a^	27.84	3.50 ± 0.20 ^a^	30.28	3.40 ± 0.10 ^a^	26.79	1.87 ± 0.08 ^b^	25.13	2.21 ± 0.15 ^b^	29.39
	bitter	5.80 ± 0.21 ^a^	41.43	5.26 ± 0.34 ^b^	42.08	4.07 ± 0.18 ^c^	35.21	5.23 ± 0.25 ^b^	41.21	3.06 ± 0.64 ^d^	41.13	2.88 ± 0.59 ^d^	38.30
	tasteless	1.56 ± 0.12 ^a^	11.14	1.39 ± 0.05 ^ab^	11.12	1.31 ± 0.07 ^abc^	11.33	1.43 ± 0.44 ^ab^	11.27	0.99 ± 0.09 ^cd^	13.31	0.84 ± 0.10 ^d^	11.17
808	umami	6.14 ± 0.22 ^a^	29.10	5.21 ± 0.16 ^b^	27.98	4.19 ± 0.07 ^c^	29.02	4.93 ± 0.34 ^b^	26.45	2.77 ± 0.18 ^d^	29.10	2.61 ± 0.19 ^d^	27.68
	sweet	4.95 ± 0.64 ^a^	23.46	4.47 ± 0.14 ^a^	24.01	3.54 ± 0.13 ^b^	24.52	4.33 ± 0.15 ^ab^	23.23	2.29 ± 0.26 ^c^	24.05	2.42 ± 0.47 ^c^	25.66
	bitter	8.48 ± 2.32 ^a^	40.19	7.15 ± 0.16 ^ab^	38.40	5.63 ± 0.34^bc^	38.99	7.67 ± 0.21 ^ab^	41.15	3.72 ± 0.38 ^c^	39.08	3.82 ± 0.20 ^c^	40.51
	tasteless	1.54 ± 0.20 ^ab^	7.30	1.79 ± 0.16 ^a^	9.61	1.09 ± 0.86 ^abc^	7.55	1.71 ± 0.23 ^a^	9.17	0.74 ± 0.44 ^bc^	7.77	0.57 ± 0.21 ^c^	6.04

Each value is expressed as the mean ± SD (n = 3) of triplicate determinations. ANOVA and Duncan test were used to analyze the significant difference among samples from different cooking methods. Means with different letters within a row were significantly different (*p* < 0.05) in different cooking methods. ST, steam treatment; BO, boil treatment; FR: fry treatment, MI: microwave treatment; HP: high-pressure treatment; UN: uncooked.

**Table 3:** Effects of different cooking methods on nucleotide levels in three kinds of *L. edodes*.

Samples	Cooking Methods	Nucleotide Levels (ug/g)		
		5′-IMP	5′-CMP	5′-AMP
0912	Uncooked	12.04 ± 0.80 ^a^	8.23 ± 0.42 ^a^	33.85 ± 2.86 ^a^
	Microwaving	9.21 ± 0.71 ^b^	6.66 ± 0.37 ^b^	24.79 ± 0.29 ^b^
	High pressure	6.53 ± 0.22 ^c^	3.00 ± 0.18 ^d^	23.49 ± 1.19 ^b^
	Steaming	5.31 ± 0.33 ^c^	5.00 ± 0.16 ^c^	18.01 ± 1.01 ^c^
	Boiling	2.36 ± 0.44 ^d^	2.61 ± 0.01 ^d^	10.24 ± 0.52 ^d^
	Frying	1.51 ± 0.09 ^d^	2.62 ± 0.04 ^d^	7.68 ± 0.71 ^d^
LM	Uncooked	12.34 ± 1.14 ^a^	11.35 ± 0.15 ^a^	47.05 ± 2.24 ^a^
	Microwaving	8.16 ± 1.60 ^b^	6.59 ± 0.96 ^c^	24.34 ± 1.27 ^b^
	High pressure	7.05 ± 0.34 ^b^	7.79 ± 0.60 ^bc^	20.37 ± 0.11 ^bc^
	Steaming	7.74 ± 0.04 ^b^	9.00 ± 0.02 ^b^	19.62 ± 0.24 ^cd^
	Boiling	2.84 ± 0.22 ^c^	3.59 ± 0.03 ^d^	19.60 ± 0.55 ^cd^
	Frying	1.81 ± 0.02 ^c^	2.28 ± 0.03 ^d^	15.16 ± 1.65 ^d^
808	Uncooked	24.01 ± 1.32 ^a^	12.13 ± 0.33 ^a^	43.41 ± 1.83 ^a^
	Microwaving	20.58 ± 1.47 ^a^	9.32 ± 0.07 ^b^	39.49 ± 0.22 ^a^
	High pressure	11.63 ± 0.69 ^c^	10.24 ± 0.49 ^b^	34.50 ± 1.53 ^b^
	Steaming	16.59 ± 0.87 ^b^	7.27 ± 0.03 ^c^	29.00 ± 1.36 ^c^
	Boiling	6.14 ± 0.06 ^d^	4.95 ± 0.54 ^d^	21.95 ± 1.92 ^d^
	Frying	6.71 ± 1.26 ^d^	2.96 ± 0.08 ^e^	18.11 ± 0.95 ^d^

Notes: 5′-AMP: 5′-adenosine monophosphate; 5′-CMP: 5′-cytidine monophosphate; 5′-IMP: 5′-inosine monophosphate; a–d: significant difference (*p* < 0.05) in different processing methods. The results were expressed as means ± SD (n = 3). ANOVA and Duncan test were used to analyze the significant difference among samples from different cooking methods.

**Table 4:** The relative content of volatile flavor compounds of *L. edodes* produced in Guizhou after different processing methods (%).

Volatile Compounds	0912					LM							808					
	BO	ST	HP	MI	FR	UN	BO	ST	HP	MI	FR	UN	BO	ST	HP	MI	FR	UN
Alcohols																		
Ethanol	0.06 ± 0.00	0.06 ± 0.00	0.05 ± 0.00	0.07 ± 0.00	0.10 ± 0.01	0.12 ± 0.01	0.06 ± 0.00	0.06 ± 0.00	-		0.05 ± 0.00	0.05 ± 0.00	-	-	0.05 ± 0.00	-	0.07 ± 0.00	0.06 ± 0.00
2-furanmethanol	-	-	1.61 ± 0.12	-	-	-	-	-	1.21 ± 0.30	-	1.49 ± 0.04	-	-	-	1.61 ± 0.01	-	2.24 ± 0.02	-
2-ethyl-1-hexanol	-	-	-	-	-	-	-	-	-	-	-	-	6.41 ± 2.85	9.11 ± 0.92	-	-	-	8.72 ± 0.09
Phenylethyl alcohol	-	-	-	-	-	10.97 ± 0.00	6.13 ± 0.01	5.98 ± 0.03	-	6.72 ± 0.02	0.51 ± 0.02	5.77 ± 0.04	-	-	5.23 ± 0.07	-	-	6.32 ± 0.07
Aldehydes																		
2-methyl-propanal	-	-	-	0.54 ± 0.01	0.76 ± 0.06	-	-	-	-	-	-	-	-	-	-	-	0.51 ± 0.00	1.09 ± 0.00
3-methyl-butanal	1.15 ± 0.01	1.16 ± 0.01	0.90 ± 0.09	1.36 ± 0.02	1.91 ± 0.14	2.18 ± 0.26	1.08 ± 0.00	1.05 ± 0.01	0.68 ± 0.17	1.18 ± 0.01	0.87 ± 0.01	1.01 ± 0.01	1.22 ± 0.04	1.16 ± 0.12	-	1.35 ± 0.00	-	0.58 ± 0.52
2-methyl-butanal	1.14 ± 0.00	1.16 ± 0.01	0.91 ± 0.07	1.36 ± 0.01	1.90 ± 0.14	2.17 ± 0.25	-	1.05 ± 0.01	-	-	-	1.01 ± 0.01	-	1.16 ± 0.12	-	1.34 ± 0.00	1.27 ± 0.01	-
Pentanal	1.14 ± 0.00	-	-	-	-	-	-	-	-	-	-	-	-	-	-	-	-	-
Hexanal	2.48 ± 0.02	2.72 ± 0.24	1.98 ± 0.15	2.95 ± 0.04	3.91 ± 0.00	4.71 ± 0.55	2.33 ± 0.00	2.27 ± 0.01	1.48 ± 0.37	2.55 ± 0.01	1.16 ± 0.72	2.19 ± 0.02	2.63 ± 0.08	2.50 ± 0.25	1.97 ± 0.02	2.91 ± 0.00	2.74 ± 0.02	2.40 ± 0.03
Furfural	-	-	1.43 ± 0.11	-	-	-	-	-	1.07 ± 0.27	-	1.68 ± 0.32	-	-	-	1.43 ± 0.01	-	-	-
5-methyl-2-furancarboxaldehyde	-	-	-	-	-	-	-	-	-	-	-	-	-	-	3.02 ± 0.03	-	-	-
Benzeneacetaldehyde	-	-	-	-	10.24 ± 0.74	-	-	-	-	-	4.82 ± 0.16	-	-	-	-	-	6.81 ± 0.06	-
N-methylpyrrole-2-carboxaldehyde	-	-	-	-	-	-	-	-	-	-	-	3.25 ± 0.02	-	-	-	-	-	-
2-phenylpropenal	-	-	-	11.38 ± 0.15	-	-	-	8.76 ± 0.04	-	-	-	8.45 ± 0.06	-	-	-	-	10.57 ± 0.09	9.25 ± 0.10
Ketones																		
Acetone	0.14 ± 0.00	0.14 ± 0.00	-	-	-	-	0.13 ± 0.00	-	-	-	-	-	-	-	-	-	-	-
2,3-butanedione	-	-	-	-	1.72 ± 0.00	-	1.02 ± 0.00	0.99 ± 0.00	0.65 ± 0.16	-	0.89 ± 0.07	0.96 ± 0.01	-	1.21 ± 0.00	0.87 ± 0.01	-	-	-
Hydroxyacetone	-	-	0.47 ± 0.00	-	-	-	-	-	0.33 ± 0.08	-	0.28 ± 0.14	-	-	-	0.44 ± 0.01	-	-	-
2(5H)-furanone	0.89 ± 0.00	0.90 ± 0.01	0.71 ± 0.05	1.06 ± 0.01	1.47 ± 0.11	1.69 ± 0.20	0.83 ± 0.00	0.81 ± 0.00	0.53 ± 0.13	0.91 ± 0.00	0.69 ± 0.02	0.78 ± 0.01	0.94 ± 0.03	0.90 ± 0.09	0.71 ± 0.01	1.04 ± 0.00	0.98 ± 0.01	0.86 ± 0.01
5,6-dihydro-2H-pyran-2-one	-	-	-	2.43 ± 0.03	3.39 ± 0.25	-	-	-	-	-	1.60 ± 0.05	-	2.16 ± 0.07	-	-	2.40 ± 0.00	-	-
4-hydroxy-2,5-dimethyl-3(2H)furanone	-	-	-	-	-	-	-	-	4.77 ± 1.20	-	-	-	-	-	-	-	-	-
2-pyrrolidinone	1.02 ± 0.00	1.03 ± 0.01	0.81 ± 0.06	1.21 ± 0.02	1.78 ± 0.01	1.93 ± 0.22		0.93 ± 0.00	0.61 ± 0.15	1.05 ± 0.00	0.79 ± 0.03	0.90 ± 0.01			0.81 ± 0.01		1.12 ± 0.01	
2,3-dihydro-3,5-dihydroxy-6-methyl-4H-pyran-4-one	-	-	10.86 ± 0.84	-	-	-	-	-	8.12 ± 2.03	-	10.64 ± 0.35	-	-	-	10.85 ± 0.09	-	14.16 ± 0.76	-
2-undecanone	25.68 ± 0.11	-	20.39 ± 1.58	-	-	-	-	-	-	-	-	3.25 ± 0.02	-	-	-	-	-	-
Acids																		
Acetic acid	0.18 ± 0.00	0.18 ± 0.00	0.14 ± 0.01	0.21 ± 0.00	0.29 ± 0.02	0.34 ± 0.04	0.17 ± 0.00	0.16 ± 0.00	0.11 ± 0.03	0.18 ± 0.00	0.53 ± 0.40	0.16 ± 0.00	0.19 ± 0.01	0.18 ± 0.02	0.14 ± 0.00	0.21 ± 0.00	0.20 ± 0.00	0.17 ± 0.00
Propanoic acid	0.55 ± 0.00	0.55 ± 0.00	0.44 ± 0.03	0.65 ± 0.01	0.91 ± 0.07	1.04 ± 0.12	-	0.50 ± 0.00	0.33 ± 0.08	-	0.43 ± 0.02	0.48 ± 0.00	-	-	-	-	-	-
2-methyl-propanoic acid	-	-	-	-	-	3.79 ± 0.92	2.63 ± 0.00	2.57 ± 0.01	1.67 ± 0.42	2.89 ± 0.01	4.17 ± 2.53	1.20 ± 0.01	-	-	-	-	-	-
3-methyl-butanoic acid	-	-	-	3.34 ± 0.05	6.66 ± 2.48	2.06 ± 0.24	2.64 ± 0.00	2.57 ± 0.01	-	3.24 ± 0.34	-	-	-	-	-	3.30 ± 0.00	-	2.71 ± 0.03
Hexanoic acid	-	-	4.22 ± 0.33	-	-	-	-	-	3.16 ± 0.79	-	-	-	-	-	-	-	-	-
Esters																		
Acetic acid ethenyl ester	1.12 ± 0.00	1.11 ± 0.02	-	-	-	-	-	-	-	-	-	-	-	-	-	-	-	-
Ethyl acetate	1.33 ± 0.01	1.34 ± 0.01	-	-	2.21 ± 0.16	2.52 ± 0.29	-	-	-	-	-	-	-	-	-	-	-	-
Butyrolactone	-	1.09 ± 0.01	0.87 ± 007	1.29 ± 0.02	-	2.06 ± 0.24	1.02 ± 0.00	0.99 ± 0.00	0.65 ± 0.16	1.11 ± 0.01	0.85 ± 0.03	0.96 ± 0.01	-	1.09 ± 0.11	1.75 ± 0.88	-	1.20 ± 0.01	1.05 ± 0.01
3-methylenedihydro-2(3H)-furanone	-	-	-	-	-	-	-	1.87 ± 0.01	-	2.10 ± 0.00	-	-	-	-	1.63 ± 0.01	-	-	-
dihydro-3-hydroxy-2(3H)-furanone	-	-	6.47 ± 0.00	-	-	-	-	-	-	-	-	-	-	-	-	-	-	-
dihydro-4-hydroxy-2(3H)-furanone	-	-	2.21 ± 0.17	-	-	-	-	-	-	-	-	-	-	-	-	-	-	-
Sulfur compounds																		
Carbon disulfide	-	-	0.48 ± 0.00	-	1.09 ± 0.08	1.10 ± 0.00	-	-	0.39 ± 0.10	-	-	-	-	-	0.52 ± 0.00	-	0.73 ± 0.01	-
Dimethyl trisulfide	-	-	-	-	-	-	6.76 ± 0.01	6.60 ± 0.03	1.61 ± 0.00	7.41 ± 0.03	-	6.37 ± 0.05	7.64 ± 0.23	7.28 ± 0.73	-	8.46 ± 0.00	7.97 ± 0.07	6.97 ± 0.08
Dimethyl sulfone	1.67 ± 0.01	1.68 ± 0.01	1.32 ± 0.10	-	2.75 ± 0.20	-	-	1.52 ± 0.01	0.99 ± 0.25	-	1.30 ± 0.04	-	-	1.68 ± 0.17	1.32 ± 0.01	-	1.83 ± 0.02	1.60 ± 0.02
(methylsulfonyl)-ethene	3.27 ± 0.01	3.29 ± 0.03	-	3.88 ± 0.05	5.41 ± 0.39	-	3.06 ± 0.00	2.98 ± 0.01	-	3.35 ± 0.01	2.55 ± 0.08	-	3.45 ± 0.11	3.29 ± 0.33	-	3.82 ± 0.00	3.60 ± 0.03	-
1,2,4,6-tetrathiepane	-	25.92 ± 0.22	20.48 ± 1.58	-	31.34 ± 2.29	-	24.09 ± 0.02	23.49 ± 0.12	15.30 ± 3.83	26.39 ± 0.09	14.75 ± 0.48	22.68 ± 0.17	27.20 ± 0.83	25.91 ± 2.61	20.46 ± 0.18	30.13 ± 0.00	-	24.81 ± 0.27
1,3-dithiolane-2-thione	-	-	-	-	-	-	-	-	24.67 ± 16.85	-	-	-	-	-	-	-	-	-
Lenthionine	35.27 ± 0.16	35.46 ± 0.30	-	41.77 ± 0.56	-	63.31 ± 4.39	32.95 ± 0.03	32.13 ± 0.16	30.93 ± 5.24	36.09 ± 0.13	27.43 ± 0.90	31.02 ± 0.23	37.21 ± 1.14	45.11 ± 6.10	27.98 ± 0.24	41.21 ± 0.00	38.81 ± 0.34	33.94 ± 0.37
Pyrazines																		
Methyl-pyrazine	-	-	1.35 ± 0.10	-	5.81 ± 0.42	-	-	-	1.01 ± 0.25	-	-	-	-	-	1.35 ± 0.02	-	-	-
2,6-dimethyl-Pyrazine	-	-	2.79 ± 0.21	-	-	-	3.28 ± 0.00	3.20 ± 0.02	2.08 ± 0.52	3.60 ± 0.01	2.73 ± 0.09	3.09 ± 0.03	-	-	2.79 ± 0.02	-	3.87 ± 0.03	-
2,3,5-trimethylpyrazine	-	-	-	-	-	-	-	-	3.85 ± 0.96	-	5.05 ± 0.17	-	-	-	-	-	-	-
Others																		
D-limonene	10.38 ± 0.05	10.43 ± 0.09	8.24 ± 0.64	11.33 ± 1.19	17.16 ± 1.25	-	9.70 ± 0.01	-	-	-	8.07 ± 0.27	9.13 ± 0.07	10.95 ± 0.33	-	-	-	-	-
n-hexane	1.20 ± 0.01	1.21 ± 0.01	0.95 ± 0.07	1.42 ± 0.02	1.98 ± 0.15	2.27 ± 0.26	1.12 ± 0.00		0.71 ± 0.18	1.27 ± 0.04	0.64 ± 0.27	1.05 ± 0.00					1.32 ± 0.01	
1-(2-furanyl)-ethanone	-	-	-	-	-	-	-	-	-	-	-	-	-	-	3.02 ± 0.03	3.82 ± 0.00	-	-
2-pentyl-furan	11.60 ± 0.05	10.58 ± 0.98	0.88 ± 0.10	13.74 ± 0.18	-	-	-	-	-	-	-	-	-	-	9.21 ± 0.08	-	-	-
2-acetylthiazole	-	-	6.17 ± 0.48	-	-	-	-	-	-	-	6.04 ± 0.20	-	-	-	-	-	-	-
Phenol	-	-	-	-	-	-	-	-	1.01 ± 0.25	-	-	-	-	-	-	-	-	-
2-methyl-phenol	-	-	2.82 ± 0.22	-	-	-	-	-	2.11 ± 0.53	-	-	-	-	-	2.82 ± 0.02	-	-	-

ST, steam treatment; BO, boil treatment; FR: fry treatment, MI: microwave treatment; HP: high-pressure treatment; UN: uncooked. The results were expressed as means ± SD (n = 3). - not detected.

## Data Availability

The data presented in this study are available on request from the corresponding author.
